# Long-Term Home-Monitoring Sensor Technology in Patients with Parkinson’s Disease—Acceptance and Adherence

**DOI:** 10.3390/s19235169

**Published:** 2019-11-26

**Authors:** Angela Botros, Narayan Schütz, Martin Camenzind, Prabitha Urwyler, Daniel Bolliger, Tim Vanbellingen, Rolf Kistler, Stephan Bohlhalter, Rene M. Müri, Urs P. Mosimann, Tobias Nef

**Affiliations:** 1 Gerontechnology and Rehabilitation Group, University of Bern, 3008 Bern, Switzerland; angela.botros@artorg.unibe.ch (A.B.); narayan.schuetz@artorg.unibe.ch (N.S.); prabitha.urwyler@artorg.unibe.ch (P.U.); tim.vanbellingen@artorg.unibe.ch (T.V.); rene.mueri@insel.ch (R.M.M.); urspeter.mosimann@insel.ch (U.P.M.); 2 ARTORG Center for Biomedical Engineering Research, University of Bern, 3008 Bern, Switzerland; 3 iHomeLab, Lucerne University of Applied Sciences and Arts—Engineering and Architecture, 6048 Horw, Switzerland; martin.camenzind@ihomelab.ch (M.C.); daniel.bolliger@ihomelab.ch (D.B.);; 4 Perception and Eye Movement Laboratory, Departments of Neurology and BioMedical Research, Inselspital, University Hospital Bern and University of Bern, 3010 Bern, Switzerland; 5 Neurology and Neurorehabilitation Center, Luzerner Kantonsspital, 6000 Luzern, Switzerland; stephan.bohlhalter@luks.ch

**Keywords:** Parkinson’s disease, body-worn sensors, ambient sensors, Accelerometer, PIR sensor, acceptance, adherence, patient monitoring, telemetry, remote sensing technology, wearable electronic devices, symptom assessment, motor disorders

## Abstract

Parkinson’s disease (PD) is characterized by a highly individual disease-profile as well as fluctuating symptoms. Consequently, 24-h home monitoring in a real-world environment would be an ideal solution for precise symptom diagnostics. In recent years, small lightweight sensors which have assisted in objective, reliable analysis of motor symptoms have attracted a lot of attention. While technical advances are important, patient acceptance of such new systems is just as crucial to increase long-term adherence. So far, there has been a lack of long-term evaluations of PD-patient sensor adherence and acceptance. In a pilot study of PD patients (N = 4), adherence (wearing time) and acceptance (questionnaires) of a multi-part sensor set was evaluated over a 4-week timespan. The evaluated sensor set consisted of 3 body-worn sensors and 7 at-home installed ambient sensors. After one month of continuous monitoring, the overall system usability scale (SUS)-questionnaire score was 71.5%, with an average acceptance score of 87% for the body-worn sensors and 100% for the ambient sensors. On average, sensors were worn 15 h and 4 min per day. All patients reported strong preferences of the sensor set over manual self-reporting methods. Our results coincide with measured high adherence and acceptance rate of similar short-term studies and extend them to long-term monitoring.

## 1. Introduction

Parkinson’s disease (PD) is the second most common neurodegenerative disorder [1]. With a mean age of onset around 60 years, PD is highly age-associated [2]. The cardinal motor symptoms of PD, bradykinesia, resting tremors, akinesia and postural instability lead to a reduction of quality of life [3,4,5]. To date, there is no cure for PD and treatment mainly aims to reduce the symptoms [2]. Most motor symptoms can effectively be treated using levodopa-based medication [6,7]. While levodopa medication significantly improves quality of life of PD patients [8] there are some challenges associated. Motor fluctuations, namely between blocking and dyskinesia (involuntary movement), can occur after prolonged exposure to levodopa which reduces treatment effectiveness [9]. Timing and dosage are challenging, and it requires precise information about the occurrence and duration of the symptoms.

Disease diagnosis and progression usually consist of consultations with medical experts in a clinical setting such as a medical office or a hospital. The Unified Parkinson Disease Rating Scale (UPDRS) is the gold standard for the assessment of PD motor and non-motor symptoms. It assesses through self-evaluation and rating and monitoring by clinicians. However, there are also some disadvantages to this method. Some motor symptoms are known to be triggered by very specific environments, such as freezing of gait (FoG), which can be triggered by walking through doorways or turning mid-walk [10]. These situations can be hard to reproduce in clinical settings. Regular visits to a hospital or clinic for symptom assessment can be a burden to patients with reduced mobility. Furthermore, strong intra-day and intra-person symptom fluctuations during the day make it challenging for medical professionals to correctly classify the severity of the PD symptoms [11]. For a better insight into motor-symptom fluctuations, another method for medical professionals to rate the long-term progress of the patient’s disease are self-reporting PD diaries, led by the patients. These PD diaries can be either in paper form or included in an app on an electronic device [12]. Patients note down the medication intake and approximate time of symptom occurrence. Patient-led symptom diaries are widely accepted and well established [13]. Advantage of these diaries are the obtained long-term information about symptoms and progress throughout the day. The main disadvantage is the necessity for patients to regularly write down their symptoms manually. This can lead to low patient adherence and satisfaction while also suffering from recall bias and thus increasing the method’s subjectiveness [14]. From both a medical point of view as well as from the patient perspective, there is thus a strong need for objective and reliable PD symptom monitoring systems that require minimal manual interaction. Additionally, a more objective profile of the patient’s fluctuations can assist the medical professionals’ decision-making process regarding medication protocol.

The usage of wearable sensors to identify movement patterns such as gait assessment [15] or body position [16] dates back over 20 years. Over the past couple of years, these sensors have not only become cheaper, but also smaller, lighter and equipped with increasingly longer battery life, making them very well-suited for long-term (e.g., several weeks) objective and automatic data collection. In the field of PD symptom detection and identification, wearable sensors have been the primary sensor type for motor-symptom detection [17,18,19,20]. However, so far, usage of wearable sensors has mostly been constrained to application in clinical settings such as research labs, hospitals or other controlled environments. Only recently, with the extension of battery life has it become feasible to develop wearable sensor-based symptom identification systems for use at home.

There have been a couple of studies looking at the usability of individual sensors as well as multi-component sensor systems in clinical and home-based applications [21,22,23]. However, only a few studies have addressed the patient’s perspective on these new sensor systems. The patients’ acceptance of such new systems and adherence to them should be a crucial part of their development [24]. Tzallas et al. evaluated a sensor set consisting of five body-worn sensors, designed to identify PD motor symptoms [25], after their sensor set reliability was evaluated in a previous study [26]. The sensor set was first evaluated over a short time period, 15 min, in a clinical setting, followed by a long time period, 8 h a day over 5 consecutive days, at the PD patients’ homes. The sensors were placed on both left and right wrist as well as both left and right ankle. The fifth sensor was placed at the hip and a data-logger was carried with a small bag. After the respective measurements, the participants gave detailed feedback about their experiences. The feedback was rated to value the acceptance of the system as satisfactory. Fisher et al. evaluated a sensor set consisting of two wrist sensors [27]. Again, the sensor set was designed to identify PD motor symptoms. The sensors were first tested in a clinical setting with 34 PD patients, then during one week at the PD patients’ homes. At home, participants wore the sensors continuously. After 7 days, the participants were asked to fill in a questionnaire regarding their experience and acceptance of the sensors. The sensor data was evaluated in [28]. Both the studies by Tzallas et al. and Fisher et al. reported good acceptance rates [25,27].

Another form of sensors that can be used in a home-based setting are ambient sensors. Ambient sensors are installed at fixed locations and no sensors must be worn by the monitored people. This reduces the burden on the measured people. These sensors have been used to monitor both healthy elderly people as well as people with mild cognitive impairment [29,30]. El Helw et al. propose an integrated multi-Sensing framework including ambient sensors to monitor Parkinson’s disease progression [31]. While body-worn sensors record body movement in detail, ambient sensors collect data about the person location and could thus be used to add additional contextual information, including things such as location-patterns or the presence in a specific room. This additional information may help to increase accuracy of body-worn sensors, without adding more of them.

In this study, we evaluate the adherence and acceptance of long-term (4 weeks) usage of an at-home sensor set. We aim to extend existing results regarding acceptance and adherence from short-term studies. The sensor set consists of three body-worn and seven ambient sensors and is built for identification of PD motor symptoms. To our knowledge, there have been no studies of comparable length involving a combination of both body-worn and ambient sensors.

## 2. Results

In this study, we evaluated the PD-patient acceptance and adherence of a set of body-worn sensors and a set of in-home ambient sensors over a four-week period. Acceptance was measured through a set of established questionnaires. The PD patients’ adherence was quantified by the relative wear time of the body-worn sensors.

### 2.1. Outcome Measure–Questionnaire

The study participants filled in two questionnaires, the SUS and an ad-hoc questionnaire. Over both questionnaires, results were weighted such that missing questions were not included. The mean SUS score was at 71.5% (SD 12.8).

**Figure 1:**
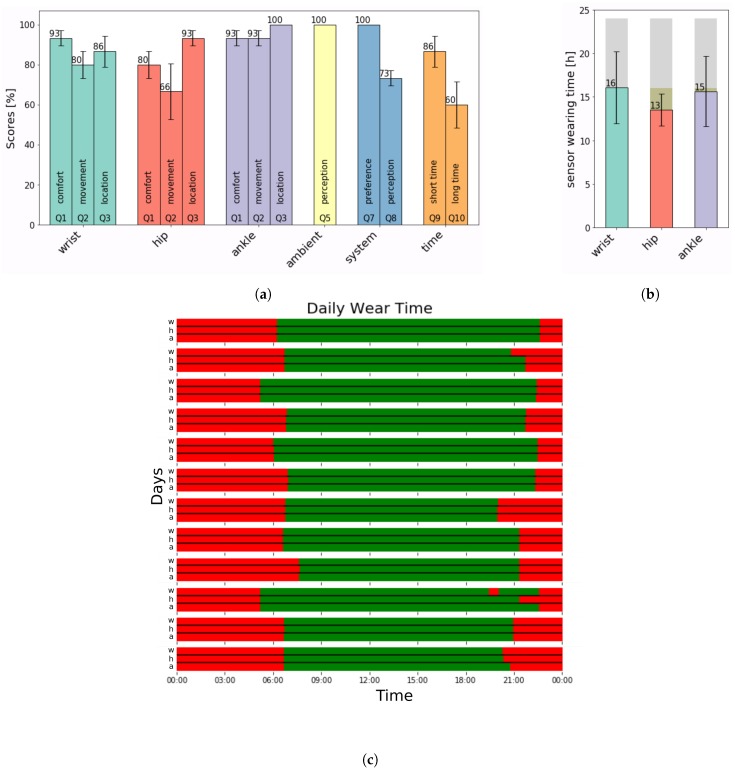
Results from acceptance and adherence evaluation. (**a**) Answer scores from the questionnaire. The question item is depicted at the bottom of each bar. Items Q1–Q3 are repeated once for every body-worn sensor. (**b**) Total wearing time of the three body-worn sensors over 4 weeks. The grey bar outlines 24 h, the yellowish bar outlines the 16 h mark. (**c**) Example of daily sensor-wearing time over consecutive 12 days. Green is wearing time; red is non-wearing time. For every day, wrist, hip and ankle sensors are listed individually.

Results for all question items Q1 through Q10, excluding the open-end questions, are presented in Figure 1a. Answers to the open-end question Q4 for all three sensor-locations, Q6 and Q11 are presented in Table 1. All participants strongly favored the sensor set over the self-reporting method and strongly disagreed with the statement to rather use the self-reporting method over the sensor set, Q7. This resulted in a 100% score favoring the sensor set over the self-reporting method. Both the dislike of the self-reporting method as well as the preference of the sensor set was confirmed verbally, on multiple occasions. Nevertheless, the participants do not feel fully at ease wearing the sensors in public, Q8, as the score of 73.3% (SD 3.8) indicates. Patients indicate high willingness to use the sensor set over short time periods, Q9, i.e., less than 2 months (86.7% SD 23.1). The patient’s willingness to use the system over longer time periods, Q10, i.e., 6 months, is lower (60% SD 34.6).

### 2.2. Analysis–Adherence

In Figure 1b, the measured sensor-wearing time of all four participants is depicted. Overall, mean sensor-wearing time is 16 h 4 min (SD 4.13) for the wrist sensor, 13 h 31 min (SD 1.83) for the hip sensor and 15 h 37 min (SD 4.04) for the ankle sensor. The *p*-value results from the paired *t*-test for every person and every sensor pair is given in Table 2. Participant P1, P2 and P4 have no significant differences in wearing times for the three sensors. A 12-days extract of the sensor-wearing time of participant P1 if given in Figure 1c. Participant P3 wore the wrist and ankle sensor also during night-time, resulting in significantly different wearing times between these two sensors and the hip sensor. Excluding the wearing time of P3, the wrist sensor is worn 13 h 50 min (SD 2.0), the hip sensor is worn 13 h 33 min (SD 2.19) and the ankle sensor is worn 13 h 34 min (SD 2.19).

### 2.3. Data Validity and Usability

In the data evaluation process, there was no noticeable loss of data. This coincides with results from previous studies performed with the same sensors [30,32]. Results from the usability test for bradykinesia, tremor and dyskinesia are given in Table 3. Since there were not enough gait abnormalities recorded during these measurements, our obtained data was compared to existing data sets and their approach on detection of gait abnormalities. Our obtained data is recorded at the same body position and with similar sampling frequency as the Daphnet data set [17].

## 3. Discussion

The strength of this study was the long measurement duration and the continuous 24 h monitoring. The relatively small number of participants is sufficient for the purpose of the long-term usability testing.

To our knowledge, this is the first long-term evaluation of PD patients’ acceptance with and adherence to home-monitoring systems. The reported acceptance and measured adherence are in accordance with past studies over shorter time periods. In the study by Tzallas et al., 12 PD patients wore a set of 6 body-worn sensors over 5 consecutive days [25]. They reported the system to be acceptable. In the study by Fisher et al., 34 patients wore 2 wrist sensors over seven consecutive days [27]. They reported high patient concordance with the body-worn sensors. Furthermore, as in our study, most patients showed a strong preference towards the body-worn sensors over the self-reporting method. However, the comfort of the sensors seems to be an important point, as the reported comfort deteriorated over time with regards to the wrist-worn sensors. This is, to a lesser extent, also reflected in our study. Out of the seven comments given in the open-end question items in Table 1, five are concerned with specific problems when wearing the sensors, e.g., trouble when changing cloths. The perceived stigma of wearing the sensors publicly is also addressed in question item Q8: “I feel uncomfortable wearing the sensors in public.” which scored at reverse-coded 73.3% (SD 3.8), meaning, the patients disagree with this statement with 73.3%. Most of the problems were addressed during our study and overall scores from Q1, Q2, Q3 are all relatively high, but the feedback indicates there is still room for improvement to integrate the sensors in every day wearing habits such that the sensors are even less noticeable and bothersome. From the patients’ side, sensor-comfort and perception is a very important point when choosing a sensor set. Integration of the sensors in the form of wearables, i.e., sensors directly integrated into everyday clothing, jewelry, watches or similar objects could be an alternative to the body-worn sensors. However, further testing would be required for evaluation and usability of these types of sensors.

Besides the body-worn sensors, a set of 7 ambient sensors plus a base station for data handling and forwarding was installed in the participants homes. None of the participants showed any concerns about these types of sensors and one person added a comment in the open-end question item Q6 that they forgot about the sensor all together. This feedback was also given on multiple occasions in oral form at the second and third meeting with the patients. These results suggest that, at least from a patient perspective, it might be a valid strategy to incorporate such ambient sensor set in PD symptom monitoring.

The reported SUS score is lower (71.8% SD 12.8) than the scores from the additional questionnaire. A reason for this discrepancy could be the more detailed questions of the additional questionnaire as opposed to the general questions of the SUS. In Bangor et al., 2009, an empirical study matching the SUS score to one of seven adjectives (Worst Imaginable, Awful, Poor, OK, Good, Excellent, Best Imaginable) concluded that the adjective ‘Good’ corresponds to a SUS score of 71.4 (SD 11.6) when rating a product [33].

During the 4 weeks of measurement, the patients were asked to use a self-reporting method to keep track of their motor symptoms. While this is a common method to observe both daily and day-to-day fluctuations, this method suffers from some disadvantages. Keeping patient adherence up for longer periods can be challenging, recall bias and diary fatigue can alter the validity of the entries [14]. This was confirmed in question item Q7, with 100% score (SD 0.0) in favor of the sensor set over the self-reporting method. Additionally, on multiple occasions during the user focus meetings as well as throughout the study, oral feedback was obtained regarding the patients’ discontent towards the self-reporting method.

The four study participants showed high commitment to the task. Mean measured wearing times are between 13.5 and 16 h per day. One participant, P3, did wear two of the three sensors also during the night. Excluding his results, the other three participants have very steady wearing times, all around 13 to 14 h per day, and low deviations. This is also supported by the paired *t*-test results presented in Table 2. There is no significant (*p* > 0.01) difference in wearing time for of the three sensors for P1, P2 and P4. The participants were not asked to wear the sensors during night-time nor during showering or similar activities, which account for 7 to 9 h per day. Considering these numbers, our measurement indicates very good participation and high potential for long-term usage of such sensor sets for PD monitoring. However, results from this study cannot automatically be generalized to study other populations. With the explained aim to digitize the self-reporting symptom recording methods, most participants expressed immediate understanding, agreement and high motivation to help. This might not be the case for other studies, and long-term adherence might differ substantially [34].

Regarding the data validity and usability, primary evaluations are satisfactory. With virtually no data loss, the technical validity of the system is confirmed. While the reported sensitivity and specificity values are lower than in the reference literature, this was to be expected, considering the noisy ground truth. In the PD symptom diary, patients fill in their dominant motor symptom per every 30-min interval. On the other hand, the studies on which the used algorithms are based did detail time measurements of the symptoms, down to the seconds. Further evaluations with patients staying in a semi-supervised environment will be needed to augment our data and further improve the classification accuracy.

## 4. Limitations

This study focused on a few participants who were followed closely during the measurement. Consequently, the individual behavior of the participants influenced the results more than the effect of the long-term measurements, i.e., the choice of one participant to wear some of the sensors during night-time influenced the result of the adherence evaluation. On the other hand, by following the four study participants closely, any concerns, thoughts and inputs by the participants were immediately taken care of, to gain the best possible insight into the sensor set usage. With technology usage ranging between laggard and early adopters, the selection of participants does not wrongly favor technology-enthusiast.

## 5. Methods

### 5.1. Materials

**Figure 2:**
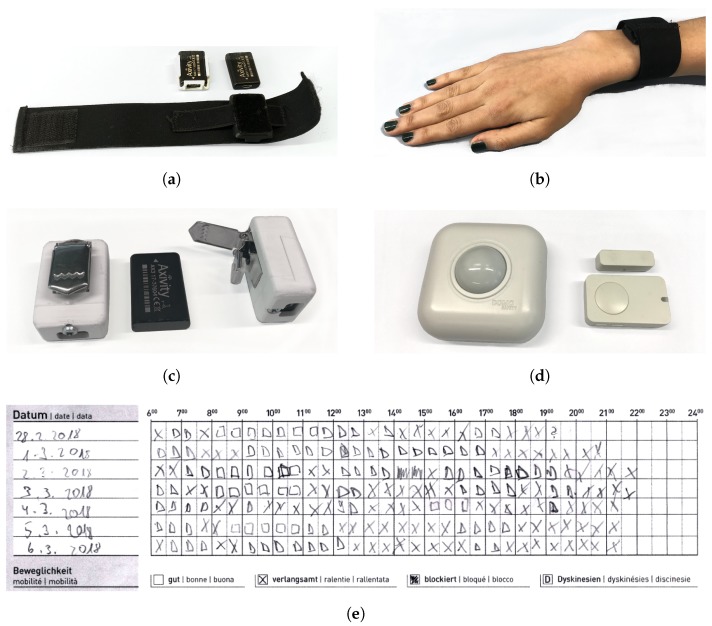
The complete sensor set with body-worn sensors as well as ambient sensors. (**a**) acceleration sensor, worn on wrist and ankle with a flexible strap and 3D-printed encasing. (**b**) The strap is simple to put on and take off, even with reduced finger or hand mobility and dexterity. (**c**)Fully enclosing sturdy shell for hip sensor. A suspender closure is used for steady fixation on the belt or the trouser seam. (**d**) The ambient sensor set consists of 5 PIR sensors (**left**), 2 two-piece magnetic door sensors (**right**) and the base station. (**e**) Example of self-reporting PD home diary.

The sensor set consists of three body-worn sensors and 7 ambient sensors. The body-worn sensors measure 3-axis acceleration (weight 11 g, l×b×h:23×32.2×7.6mm, Axivity AX3 [35]) that can record continuously at 100 Hz up to 14 days with acceleration range of ±8 g and up to 13 bit resolution. This sensor was chosen for its long battery run time, small weight and past usage in other studies [27,28,32]. One acceleration sensor each is placed on wrist and ankle with dominant motor symptom using a flexible strap and hook-and-loop closure as depicted in Figure 2a,b. The sensor-positions were selected based on existing studies [17,20,23,25] with the aim to detect as many of the cardinal motor symptoms and side effects as possible, bradykinesia, tremor, dyskinesia, gait abnormalities and postural instabilities. The material of the flexible straps for wrist and arm fixture were selected for comfort (soft touch, non-itchy) and comply with sanitary standards. The third acceleration sensor is placed on the hip using a 3D-printed casing and suspender closure as is depicted in Figure 2c.

The flexible straps used for fixating the body-worn sensors as well as the 3D-printed casing for the hip sensor were continuously tested and adapted for best usability both in the pre-study phase as well as during the study. The 3D-casing for the hip sensor started as a small lightweight skeleton-like design with a simple clip-on closure but was changed to a fully enclosing sturdy shell with a stronger suspender closure after the sensor got caught in a jacket after two days of wearing.

The set of ambient sensors consists of 5 passive infrared (PIR) sensors and two two-piece magnetic door sensors (DomoCare^®^, DomoSafety [36]) as shown in Figure 2d. The PIR sensors are placed inside the home of the PD patient, covering kitchen, bathroom, bedroom, entrance and living room, usually placed at an elevated position such that the presence of the patients is detected easily. The two door sensors are placed on the entrance door and on the fridge door. The 7 ambient sensors are equipped with long lasting batteries and do not need to be connected to another power source. The PIR sensors register movement with 0.5 Hz. The door sensors register each opening and closing of the corresponding door. A base station, plugged into a power source, communicates with all ambient sensors through the ZigBee protocol. The base station sends all received sensor data to a server through UMTS communication. Please note that since PIR sensors cannot differentiate between patients and family members, we have selected only participants living in single households.

### 5.2. User Focus Meetings

To take the perspective of technicians, clinicians, therapists as well as patients into account, four user focus meetings were held. In the first two meetings, the number of body-worn sensors was decided upon, together with technicians, researchers and PD patients. In the subsequent meetings, exact body position of the sensors were discussed with prospective study participants. These discussions also included sensor fixation for the body-worn sensors. Results from these meetings influenced the development process of the fixtures discussed in the previous Section 5.1.

### 5.3. Subject Recruitment

The study design was carried out in accordance with the current version of the Declaration of Helsinki and approved by the Ethics Committee of the Canton of Bern, Switzerland (KEK-Nr. 406/16). The participants were recruited by doctors and physical therapists at the neurorehabilitation unit of a regional hospital. Inclusion criteria were the ability to fill in the PD symptom diary independently or with the help of primary caregivers if limitations in finger-dexterity occurred. There was no exclusion criterion based on their technical familiarity. The study procedure and all devices were explained to the participants and a written informed consent was obtained prior to participation. Four adults (2 male, 2 female; age 65–70; disease duration 10–14 years, UPDRS 11–49) were included in this study. Their technical familiarity ranged from laggard to early majority [37]. Patients expressed different disease stages and different symptom distributions. Participants’ medication intake for PD related ailments were recorded in the PD symptom diary. All study participants were instructed to wear the sensors during waking hours. They were not required to wear the sensors during the night nor when taking a shower, bathing or similar activities but were not prohibited from doing so.

### 5.4. Data Collection

After the participants filled out the informed consent, a first meeting was set at their homes. At the first meeting, the ambient sensors were installed in the patients’ homes and the body-worn sensors were handed to the patients with detailed instructions. Patients wore the body-worn sensors during waking hours. During all 4 weeks of measurement, the patients kept their PD home diary. An extract is depicted in Figure 2e. The second meeting was at the beginning of the third week when the body-worn sensors were recharged. This meeting was also used to obtain general oral feedback from the participants. The third meeting was at the end of the fourth week. The body-worn sensors were collected, and the ambient sensors uninstalled. After the last meeting, the patients were given two questionnaires to fill out.

The first questionnaire-based outcome measure was self-reported system usability, evaluated by the System Usability Scale (SUS) [38]. The SUS is a generalized usability measure which collects users’ subjective perception of interaction with different interfaces. Two usability aspects are important: the patients’ ability to handle the sensors and their willingness to use the sensors on a day-to-day basis. The SUS is a Likert-scale-like questionnaire with ten items on a 5-point scale ranging from 1 (strongly disagree) to 5 (strongly agree). There are five items with positively formulated questions and five items with negatively formulated questions. The SUS takes three different usability criteria into account: effectiveness, efficiency and satisfaction. The item scale is weighted such that resulting points range from 0 to 100 with 100 being the best outcome and 0 being the worst outcome. We used a validated German translation of the SUS [39]. The second questionnaire is based on work by Fisher et al. [27]. They tested a set of body-worn sensors for PD motor-symptom tracking during one week in a home-based setting and evaluated the participants’ experiences through a questionnaire. All question items used in our questionnaire are presented in Table 4. As with the SUS, all question items are rated on a 5-point scale from 1 (strongly disagree) to 5 (strongly agree). In the evaluation, all negatively formulated question (Q2, Q5, Q7 and Q8) are reverse coded by subtracting their value from 6. Resulting values are added and weighted such that the resulting points range from 0 to 100, with 100 being the best and 0 the worst outcome.

The questionnaire is split into four parts. The first and second part inquire the sensor-individual acceptance by the study participants. In the first part, question items Q1, Q2, Q3 and Q4 are posed for each of the three body-worn sensors, with adjusted sensor and body-placement description, ankle, wrist, hip. In the second part, two question items, Q5 and Q6, inquire the sensor-individual acceptance of the ambient sensors. In the third part, two question items, Q7 and Q8, inquire the preferences of the two systems, sensor set vs. self-reporting method. The last part consists of two question items, Q9 and Q10, regarding short- and long-term acceptance of the sensor set. At the end, there is a general open-end question, Q11, for any comments not covered by the previous questions.

#### 5.4.1. Analysis

The main target of the sensor set is the replacement of the PD home diary or other self-reporting methods by a better accepted, more reliable system. Loss of data, as a result of technical or human failure or due to non-adherence, is an objective measure of the system reliability and usability. Technical reliability depends heavily on the chosen sensors and must be evaluated for each new sensor. Both chosen sensor types, wearable and ambient, have been used extensively in the past for other studies or commercial use [30,32,36]. The focus here is on the sensor-independent parts, the patient compliance and adherence in conjunction with a given sensor set.

#### 5.4.2. Adherence

To quantify the adherence, data from the body-worn sensors was evaluated and the actual wear time is calculated. The employed algorithm as well as the used threshold is based on work by Doherty et al. [32]. For every 5 s interval $T_{i}$, the standard deviation $\sigma_{s,i}$ over the data $s_{i}$ is computed on all three axes x,y,z. If $\sigma_{s,i}$ is below a lower threshold $L_{th}=13$ mg for all three axis x,y,z, this 5 s interval $T_{i}$ is classified as ‘non-wearing’. To account for accidental movement of the non-wearing sensors or periods of very low movement, e.g., napping, watching TV, the obtained ‘wearing’/’non-wearing’ times are smoothed and filtered. Only consecutive non-wearing time intervals of at least 30 min length are considered. Short state changes from non-wearing to wearing or the other way around of less than 5 min are omitted.

#### 5.4.3. Statistical Evaluations

The wearing times per sensor for every person are evaluated statistically. The daily wearing times of the three sensors are considered repeated samples per person; they are thus analyzed using a dependent *t*-test for paired samples. The significance level for *p*-values is set at 0.01. The Python/Scipy software environment is used for the calculation of the statistics results and the adherence computation.

#### 5.4.4. Data Validity and Usability

Obtained data from the wearable sensors are checked for data integrity and completeness, using methods developed by Doherty et al. [32]. Usability of data was evaluated using existing algorithms that have been tested in controlled environments. We have tested, whether the selected algorithms are applicable to our obtained data. The following four major motor symptoms have been taken into account: Bradykinesia, tremor, gait abnormalities and dyskinesia as the major side effect of the levodopa medication. For every motor symptom, the relevant algorithms were applied to the data, and feature vectors were generated. For Bradykinesia, features as described by Salarian et al. were extracted from the data [20]. For Tremor, features as described by Rigas et al. were extracted from the data [18]. For dyskinesia, features as described by Patel et al. were extracted from the data [19]. Based on the ground truth, the PD symptom diary, a bagging classifier was trained in a 10-fold cross-validation setting. The results were validated on a test set, excluded from the training. For gait abnormalities, the data structure was compared to similar data on which gait classification has been performed successfully [40].

## Figures and Tables

**Table 1:** Comments to the open-end questions Q4, Q6 and Q11. All answers are translated from German.

Open-End Questions	
Q4 (wrist):	“When changing clothes, like jumpers or jackets, the [wrist] sensor was a bit disturbing”.
Q4 (ankle):	“When changing trousers, the [ankle] sensor was a bit disturbing”.
Q4 (hip):	“In winter, the sensor is less disturbing as compared to when light clothes are worn.
	I wore the [hip] sensor at the front. On the back it was more disturbing”.
	“When using the toilet, it was bothersome”.
Q6:	“I forgot about the [ambient] sensors”.
Q11:	“Not in summer”.
	“I enjoyed participating in this study”.

**Table 2:** In this table, the *p*-values for the paired *t*-test for every participant and every two sensors are depicted.

*p*-Values	P1	P2	P3	P4
wrist-hip	0.07	0.46	<0.01	0.02
wrist-ankle	0.30	0.14	0.01	0.04
hip-ankle	0.82	0.55	<0.01	0.13

**Table 3:** Classification results, based on preliminary usability tests.

Motor Symptom	Sensitivity	Specificity	Precision
Bradykinesia	69.8%	68.3%	70.1%
Dyskinesia	69.0%	72.2%	72.5%
Tremor	74.2%	69.7%	70.8%

**Table 4:**
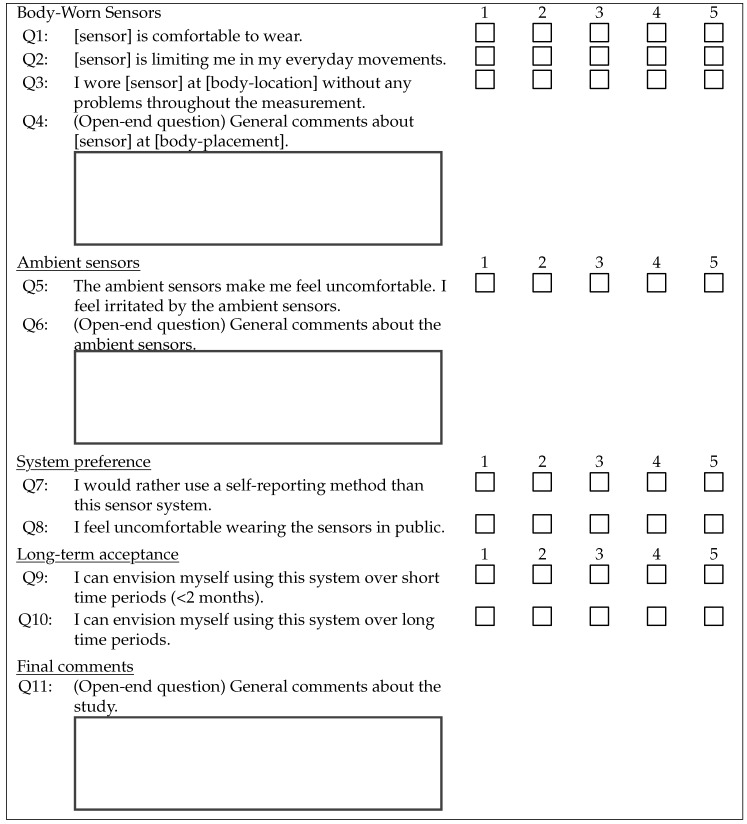
Questionnaire items additional to the SUS. In the evaluation, all questions are rated on a 5-point scale ranging from 1 (strongly disagree) to 5 (strongly agree). The negatively formulated questions Q2, Q5, Q7 and Q8 are reverse coded by subtracting their value from 6 in the evaluation.

## Abbreviations

The following abbreviations are used in this manuscript:

PD	Parkinson’s disease
UPDRS	Unified Parkinson Disease Rating Scale
H&Y	Hoen & Yahr
SD	Standard deviation
PIR-sensor	Passive Infrared Sensor
FoG	Freezing of Gait
SUS	System Usability Scale
